# Supplementary material for: Nuclear Factor-κB Overexpression is Correlated with Poor Outcomes after Multimodality Bladder-Preserving Therapy in Patients with Muscle-Invasive Bladder Cancer
Source: J Clin Med. 2019 Nov 13;8(11):1954. doi: 10.3390/jcm8111954 (PMC6912291; doi:10.3390/jcm8111954)

Table S1. A list of antibodies used for immunohistochemistry

Antibody		Clone	Company	Dilution
**p53**		DO-7	Roche Ventana, Oro Valley, AZ, USA	Ready-to-Use
**p16**		E6H4	Roche Ventana, Oro Valley, AZ, USA	Ready-to-Use
**EGFR**		31G7	Bio SB, Santa Barbara, CA, USA	1:50
**Her-2**		4B5	Roche Ventana, Oro Valley, AZ, USA	Ready-to-Use
**NFκB (p65)**		F-16	Santa Cruz Biotechnology, Santa Cruz, CA, USA	1:200
**E-cadherin**		EP700Y	Thermo Fisher Scientific, Waltham, MA, USA	1:50
**MMP9**		rabbit polyclonal	Bio SB, Santa Barbara, CA, USA	1:100
**MRE11**		12D7	Abcam, Cambridge, MA, USA	1:200
**PD-L1**		SP142	Roche Ventana, Oro Valley, AZ, USA	1:50
**MMR proteins**	**MLH1**	M1	Roche Ventana, Oro Valley, AZ, USA	Ready-to-Use
	**PMS2**	EPR3947		
	**MSH2**	G219-1129		
	**MSH6**	44		

NFκB: Nuclear factor-kappa B; EGFR: Epidermal growth factor receptor; MMP9: matrix metalloproteinase-9 (MMP9); MRE11: meiotic recombination 11 homolog; PD-L1: programmed death-1 ligand

Table S2. The scoring criteria of immunohistochemical stained biomarkers

Biomarkers	Scoring criteria	Definition of positive staining
**NFκB (p65)**	score 0: negative; score 1: faint, equivocal; score 2: moderate; score 3: strong	Score 1-3
**p16**	score 0: negative; score 1: minimal, <1%; score 2: focal, ≥1%, <10%; score 3: partial, ≥10%, <50%; score 4: diffuse, ≥50%	Score 2-3
**p53**	score 0: negative, <1%; score 1: focal, ≥1%, <10%; score 3: partial, ≥10%, <50%; score 4: diffuse, ≥50%	Score 0 or Score 4
**EGFR and Her-2**	score 0: no staining or incomplete membrane staining that is faint/barely perceptible and within ≤10% of tumor cells; score 1: weak, incomplete membranous staining that is faint/barely perceptible and within >10% of tumor cells; score 2: equivocal or indeterminate staining including circumferential membrane staining that is incomplete and/or weak/moderate and >10% of tumor cells or complete and circumferential membrane staining that is intense and within ≤10% of tumor cells; score 3: circumferential membrane staining that is complete, intense and >10% of tumor cells	Score 2-3
**E-cadherin**	score 0: negative; score 1: faint, equivocal; score 2: moderate; score 3: strong	Score 2-3

**MMP9**	Based on percentage of cytoplasmic staining in the tumor cells and evaluated at a 10%-interval; focal reactivity less than 10% was recorded at a 1%-interval	>0%
**MRE11**	scored on a continuous scale with at least 1000 tumor cells at three or more separate spots counted for each case, and the median was considered as low	>median
**IC of PD-L1**	scored as percentage of tumor area; score 0: <1%; score 1: ≥1%, <5%; score 2: ≥5%, <10%; score 3: ≥10%	Score 1-3
**TC of PD-L1**	scored as percentage of PD-L1-expressing tumour cells; score 0: <1%; score 1: ≥1%, <5%; score 2: ≥5%, <50%; score 3: ≥50%	Score 2-3
**MMR proteins (PMS2/MLH1/MSH2/MSH6)**	loss of staining was designated as complete loss of nuclear staining in all of the tumor nuclei	>0%

NFκB: Nuclear factor-kappa B; EGFR: Epidermal growth factor receptor; MMP9: matrix metalloproteinase-9 (MMP9); MRE11: meiotic recombination 11 homolog; PD-L1: programmed death-1 ligand; IC: immune cell; TC: tumor cell; MMR: mismatch repair.

Table S3. Univariate and multivariate analyses of LPFS, DMFS, and OS on clinical prognostic factors and IHC staining expression levels of molecular markers. Unfavorable group: hydronephrosis/hydroureter and/or pelvic nodal involvement; Favorable group: other than unfavorable group; NFκB: Nuclear factor-kappa B; EGFR: Epidermal growth factor receptor; MMP9: matrix metalloproteinase-9 (MMP9); MRE11: meiotic recombination 11 homolog; PD-L1: programmed death-1 ligand; IC: immune cell; TC: tumor cell; MMR: mismatch repair; 3y/5y: three-year/five-year.

Variable		LPFS				DMFS				OS			
		Univariate		Multivariate		Univariate		Multivariate		Univariate		Multivariate	
		3y/5y (%)	*p*-value	HR (95% CI)	*p*-value	3y/5y (%)	*p*-value	HR (95% CI)	*p*-value	3y/5y (%)	*p*-value	HR (95% CI)	*p*-value
**Sex**	Male	80/76	0.20	1.6	0.36	92/82	0.10	2.6	0.20	92/87	0.11	1.8	0.42
	Female	74/52		(0.6–4.8)		68/56		(0.6—11)		80/66		(0.4—7.4)	
**Age (years)**	≤70	83/78	0.13	2.0	0.29	92/86	0.05	0.8	0.86	91/84	0.05	0.5	0.46
	>70	71/53		(0.6—6.9)		72/65		(0.2-—4.2)		76/65		(0.1–2.7)	
**Clinical T stage**	T2	75/64	0.72	2.9	0.11	79/74	0.91	5.5	0.10	86/78	0.93	4.0	0.15
	T3	82/73		(0.8—6.9)		90/66		(0.7—42)		82/65		(0.6—26)	
**Risk factors**	Favorable group	75/68	0.43	1.3	0.69	88/81	0.04	0.5	0.34	87/81	0.05	0.5	0.45
	Unfavorable group	87/58		(0.3–4.9)		75/50		(0.1–2.3)		73/49		(0.1–2.6)	
**NFκB (p65)**	Negative (≤0)	87/66	0.05	0.1	0.001	92/85	0.03	0.1	0.01	91/82	0.04	0.1	0.014
	Positive (>0)	60/53		(0.04–0.5)		67/53		(0.02–0.6)		73/60		(0.03–0.7)	
**p16**	Negative (score 0–1)	66/52	0.01	18	0.002	75/62	0.10	5.2	0.10	83/69	0.11	3.4	0.18
	Positive (score 2–3)	87/77		(3.0–110)		93/85		(0.8–36)		93/84		(0.6–21)	
**p53**	Negative (score 0 or 4)	67/53	0.09	8.3	0.005	76/61	0.08	5.3	0.09	79/70	0.08	5.5	0.09
	Positive (score 1–3)	93/80		(1.9–37)		93/86		(0.8–35)		93/86		(0.8–38)	
**EGFR**	Negative (score 0–1)	78/66	0.99			90/84	0.16			95/83	0.18		
	Positive (score 2–3)	77/57				68/57				76/68			

**Her-2**	Negative (score 0–1)	83/64	0.38	79/70	0.40	86/74	0.41
	Positive (score 2–3)	66/56		91/79		92/79	
**E-cadherin**	Negative (score 0–1)	67/67	0.74	67/67	0.53	83/67	0.58
	Positive (score 2–3)	80/66		85/77		91/81	
**MMP9**	Negative (≤0)	77/70	0.31	83/75	0.83	67/80	0.93
	Positive (>0)	82/50		91/82		91/76	
**MRE11**	Low (≤median)	77/68	0.53	86/77	0.50	86/78	0.43
	High (>median)	78/56		77/57		82/75	
**PD-L1 IC**	Negative (≤0)	74/56	0.55	87/75	0.96	91/82	0.97
	Positive (>0)	82/70		77/77		82/76	
**PD-L1 TC**	Negative (≤5%)	83/58	0.55	85/76	0.93	89/74	0.99
	Positive (>5%)	67/67		75/75		83/75	

Figure S1. The images of Western blot showing the decreased p50 and p-p65 proteins of the knick-down cells

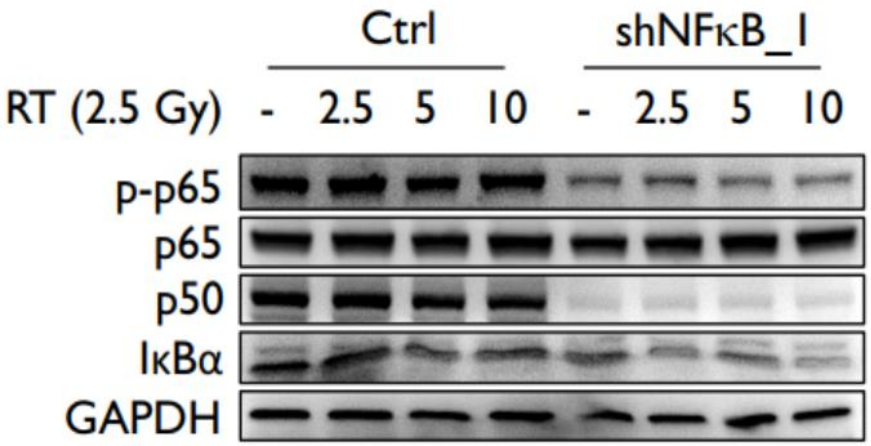

Figure S2. Representative microphotographs of immunohistochemical staining of positive and negative NFκB and p16 at magnifications of x200 in patients with bladder cancer undergoing trimodality bladder-preserving therapy.

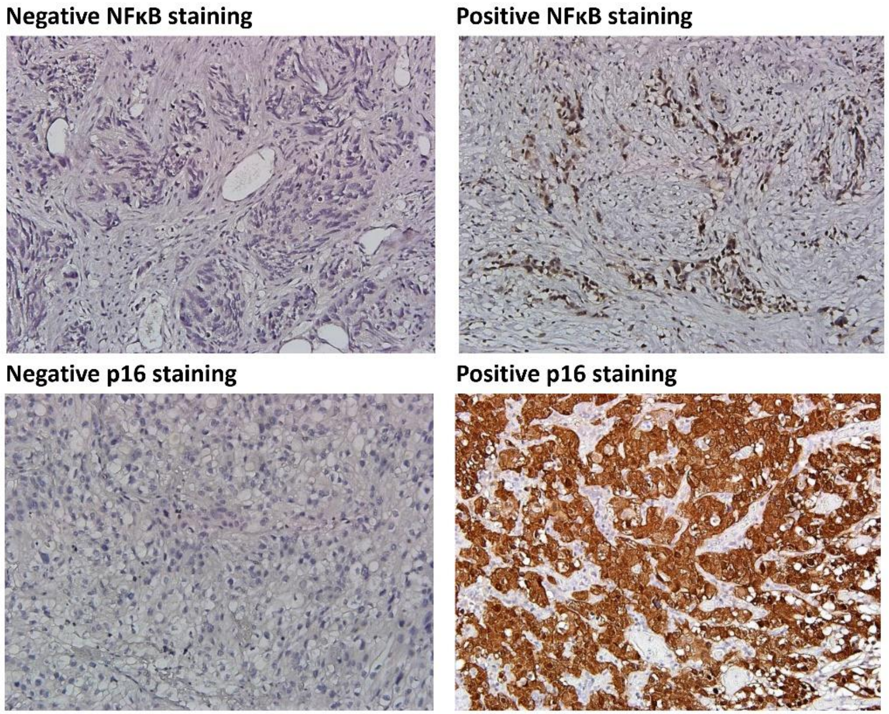

Figure S3. Kaplan-Meier curves of (A) distant metastasis-free survival (DMFS) and (B) overall survival (OS) stratified by the immunohistochemical staining of p16.

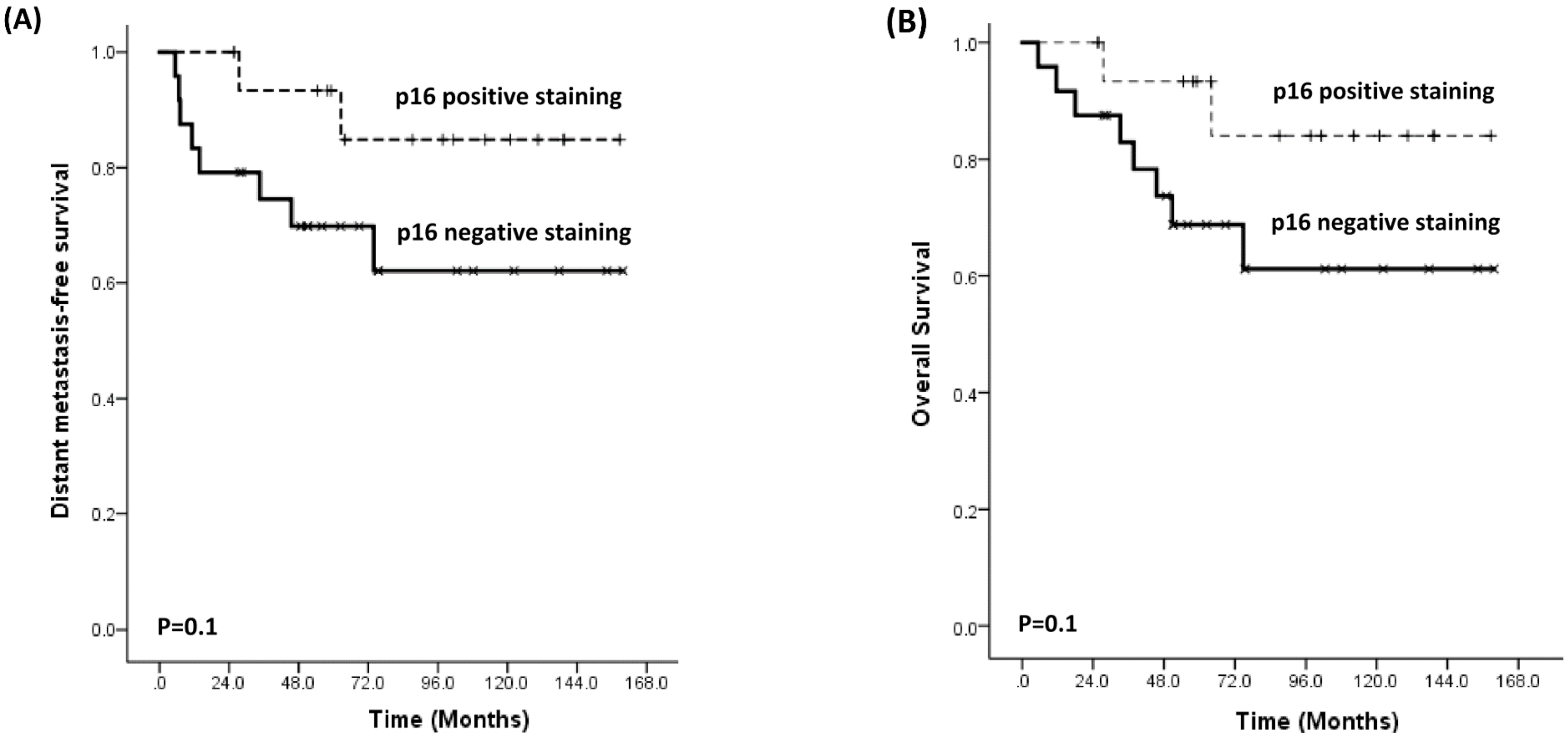

**p16 positive staining (N)**	17	15	13	8	7	4	1	0
**p16 negative staining (N)**	24	18	14	9	6	3	1	0

**p16 positive staining (N)**	17	15	12	9	8	4	1	0
**p16 negative staining (N)**	24	20	15	9	5	3	1	0

Figure S4. Representative microphotographs of immunohistochemical staining of positive and NFκB in pre-treatment and recurrent bladder tumors at magnifications of x200.

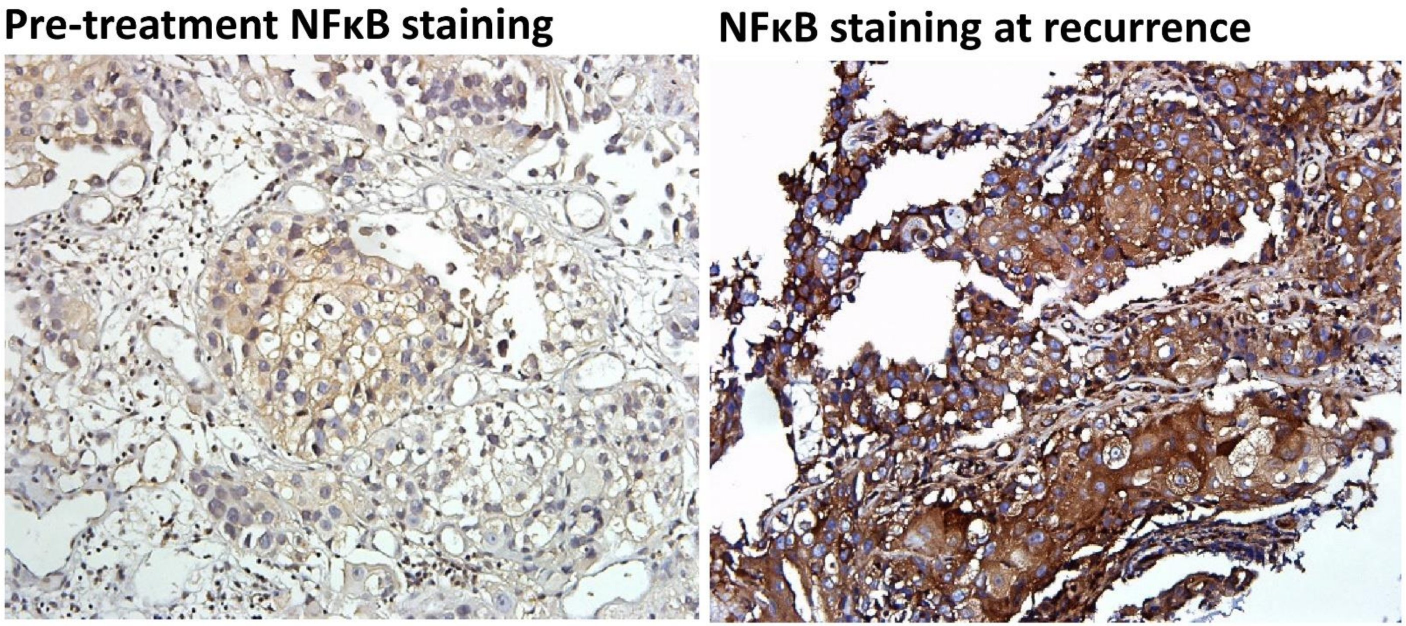

Figure S5. Concentrations (pg/ml) of IL-1β by ELISA at 24hr after 10-Gy or 20-Gy irradiation (RT) of MB49 wild type (WT) cells and MB49 NFκB knock-down (KD) cells.

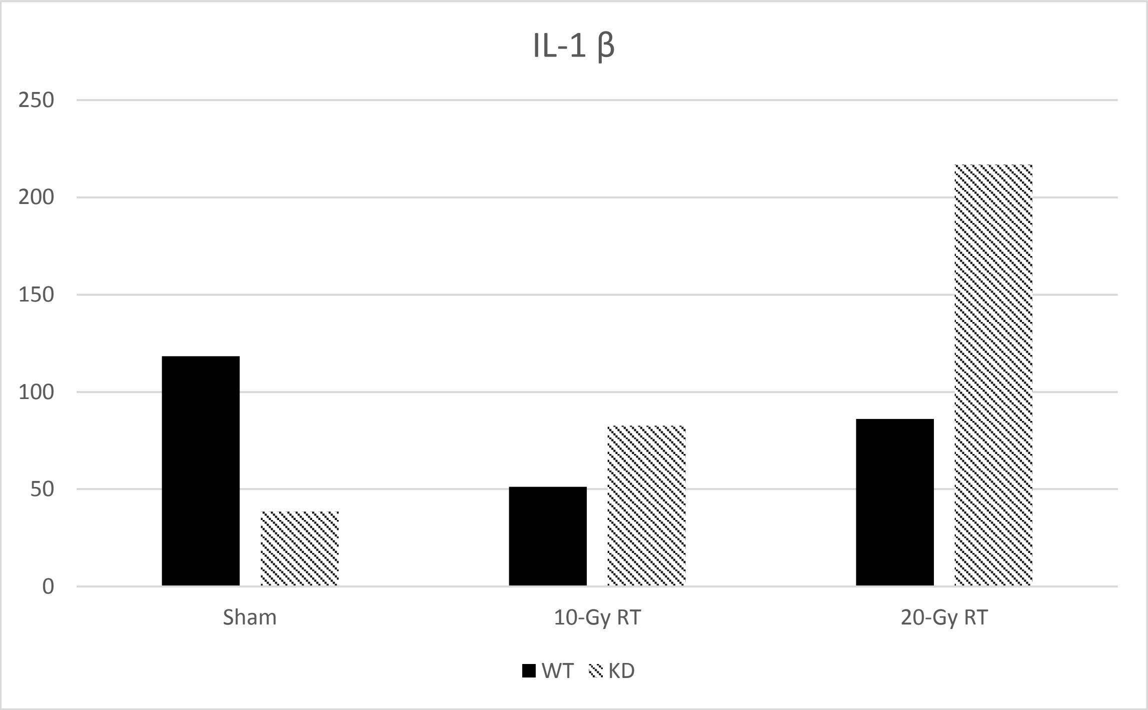

Figure S6. Colony formation assays of irradiated (0 or 10 Gy) MB49 and T24 cancer cells treated with or without the added recombinant IL-1β protein (1 ng/ml or 2 ng/ml).

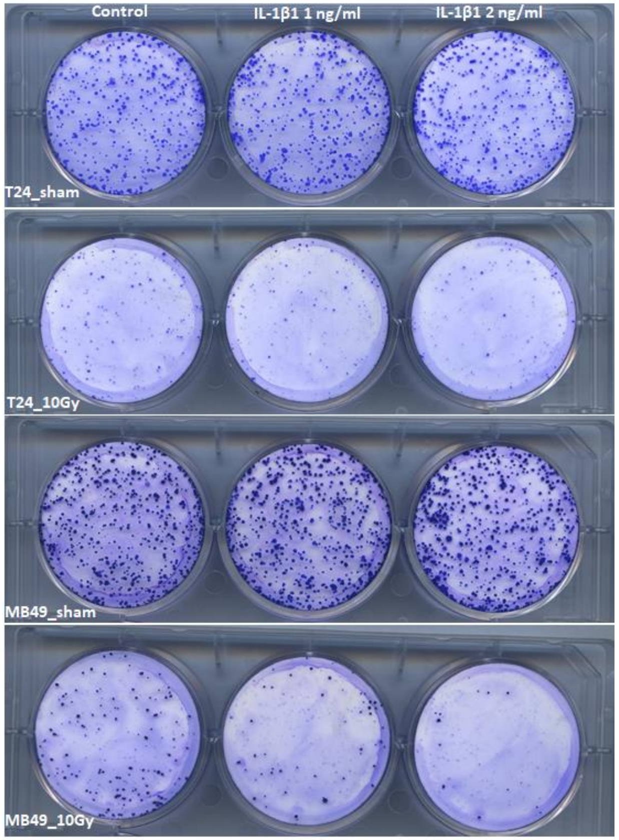

Supplement: Supplementary file 1 [file jcm-08-01954-s001.pdf]
